# Role of Ultrafast Electron-Thermal-Phonon Interactions in High Harmonic Generation and Dephasing from Graphene

**DOI:** 10.1021/acs.jpclett.6c01354

**Published:** 2026-07-04

**Authors:** Adam Herling, Ofer Neufeld

**Affiliations:** † 26747 Technion- Israel Institute of Technology, Schulich Faculty of Chemistry and Faculty of Physics, Haifa 32000036, Israel; ‡ 26747 Technion- Israel Institute of Technology, Schulich Faculty of Chemistry, Haifa 32000036, Israel

## Abstract

High harmonic generation (HHG) in solids arises when intense lasers drive attosecond-to-femtosecond electron dynamics within solid bands, causing high-energy emission. The main physical players in HHG are the electrons and photons, which are commonly thought to dictate HHG spectra. However, solids also host ubiquitous phonons that are usually relevant on longer timescales, and have therefore largely been neglected. It remains unclear if/how phonons partake in HHG and in dephasing of the electron dynamics, which has been very recently proposed. We theoretically study HHG in graphene by including optical phonons in the static limit, where the lattice is frozen on the electronic timescale and HHG is computed by sampling thermally occupied phonons and ensemble-averaging. We show that in graphene: (i) Optical-phonons strongly suppress HHG by coupling to interband currents and causing harmonic phase scrambling (destructive interference). This could potentially explain lack of experimental observation of HHG above ∼3 eV from graphene. (ii) HHG yields become temperature-dependent, though in graphene this dependence is weak due to phonon modes being frozen-out. (iii) Thermal phonons dephase interband coherences in a rate equivalent to *T*_2_ ∼ 5.7 fs, which becomes slower with weaker laser power. This timescale is substantially faster than *e* – *e* scattering, suggesting thermal phonons dominate decoherence in strong-fields. (iv) Phonons smoothen HHG ellipticity-dependent curves, better matching experiments. Remarkably, these effects are timescale-independent and arise in the static picture of electron–phonon interactions that is valid at attosecond timescales. Our results shed light on the dephasing time problem in HHG and should be transferable to other materials and processes as well (e.g., Floquet physics and photocurrents), motivating novel spectroscopies of phonon dynamics.

## Graphical abstract

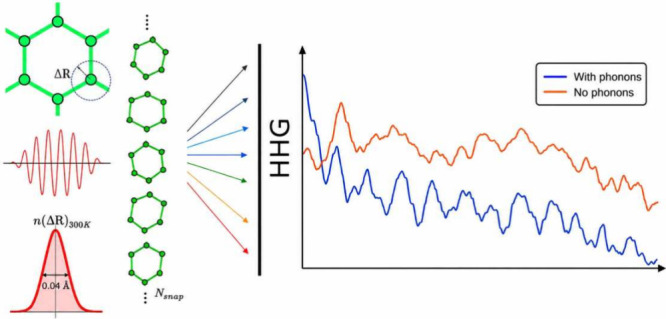

Since its seminal observation in 2011,^1^ high harmonic generation (HHG) in solids has been heavily studied both theoretically and experimentally.^2–4^ One main motivator for the field is the potential application of HHG for probing material properties in- and out-of-equilibrium. Since the main HHG mechanism arises from a combination of intraband emission and interband coherences,^3,5^ the process is directly sensitive to the band structure and curvature, as well as to electronic ultrafast band occupation dynamics. This has been applied for a variety of spectroscopies, including reconstructions of bands,^6–8^ Berry curvatures,^8–10^ coherent phonon dynamics,^11–15^ valley occupations,^16–20^ etc. Recent reports also connect HHG emission to Floquet band dressing.^17,21–24^

One main gap in current theory of HHG is that it is understood purely at the electronic level, with electrons interacting only with laser photons (classical or quantum.^25,26^ Several works in recent years also allow electron–electron (*e*–*e*) interactions at various levels,^27–33^ but broadly speaking, consideration of other particles in HHG mechanisms has been limited. This is in contradiction to modern theory of solid-state, where phonons are ubiquitous and omnipresent, having dominant contributions to various phenomena from heat capacity to superconductivity. Most recently, coherently pumped phonons were probed in HHG,^11–15^ where electron–phonon interactions were included. However, in these works the phononic coherence was essential, and the usual regime where phonons are populated thermally and incoherently is poorly understood. This open question is connected to another major challenge - the problem of HHG ultrafast dephasing times (*T*_2_). It has been established across multiple works that extremely short phenomenological dephasing times (down to few femtoseconds or less) need to be used in simulations to obtain ‘clean’ spectra matching experiments (e.g., see refs. 34–37 ). These timescales grossly mismatch with expectations based on separate measurements of coherence times that often yield many tens or hundreds of femtoseconds,^38,39^ even in strong fields.^17,19,40^ Several different works proposed various solutions to this conundrum, including propagation and macroscopic physics,^41^ quantum-optical effects,^42^ dynamical electron–phonon coupling,^43^ and Brillouin-zone (BZ) averaged dephasing due to electron–phonon interactions.^37,44^ Generally, the idea that phonons might be in-charge of ultrafast dephasing has been slowly taking hold, including an earlier work that connected dephasing with temperature effects in toy models^45^ (though there HHG yields were not greatly impacted and mostly the cutoff was explored). Still, the mechanism that allows phonons to act on such fast timescales remains vague. BZ-averaging might lead to faster dephasing in some light-matter regimes,^37^ but in large-gap systems that mechanism is unlikely. It is also not clear if optical or acoustic phonons are in charge.^43^ We further note that while working on this manuscript, we became aware of several other recent reports that deal with similar questions but from slightly different angles.^46–50^

Here we theoretically explore HHG from graphene, a prototypical 2D material used in many HHG and ultrafast experiments.^51–55^ We simulate HHG using two-band semiconductor Bloch equations (SBE) coupled to graphene optical in-plane phonon modes (transverse and longitudinal). We thermally occupy these modes and sample their quantum distribution, obtaining the laser-driven current via ensemble averaging (as arises in statistical mechanics approaches such as those recently employed in liquid HHG^56–58^ ). This scheme effectively mimics the experimental conditions where HHG is contributed by emission from large crystal volumes with numerous unit cells with random phononic phases. Using this technique, we study HHG temperature dependence, ultrafast dephasing, and dependence on the laser parameters. We find that HHG from graphene is strongly suppressed above ∼3 eV. The suppression is largely temperature-independent due the graphene phononic energy scales, but can lead to temperature dependence in other solids depending on their phononic bands. This result may contribute to the explanation of lack of experimental observations of harmonics above ∼3.1 eV^51–55^ in graphene, despite numerous simulations consistently predicting higher-energy emission. We study the mechanism behind this effect and show that it arises from optical phonons “scrambling” HHG phases, inducing strong destructive interferences (while perturbative harmonics remain phase-synced). This effect arises only in the interband emission channel. This result is remarkable considering that the timescales of phononic motion are completely irrelevant - the static lattice displacements cause phase scrambling in a timescale-independent mechanism that should also appear in attosecond experiments. We further analyze the dephasing dynamics of the interband coherences, finding decays equivalent to *T*_2_ ∼ 5.7 fs, indicating that in HHG and strong-field physics electron–phonon scattering is the dominant decoherence channel rather than *e* – *e* scattering. Interestingly, this decay time becomes slower as the laser power reduces. Lastly, we show that optical phonons can modulate the HHG ellipticity dependence, posing an interesting channel for probing temperature-dependent effects and phononic occupations on ultrafast timescales.

Let us begin by describing our methodological approach and employed formalism, starting with the standard phonon-free case. In absence of interactions with phononic degrees of freedom (DOF), electrons in graphene are described by a second-order nearest-neighbor tight binding (TB) Hamiltonian of the form

$$H_{0}\left(k\right)=\left[\begin{array}{ll} t_{2}\underset{j}{\sum }e^{-iv_{2,j}\cdot k} & t_{1}\underset{j}{\sum }e^{-iv_{1,j}\cdot k}\\ t_{1}\underset{j}{\sum }e^{iv_{1,j}\cdot k} & t_{2}\underset{j}{\sum }e^{-iv_{2,j}\cdot k} \end{array}\right]$$ 1

where *t*_1,2_ are first and second neighbor hopping terms, respectively, **v**_1,*j*_ and **v**_2,*j*_ are the first and second neighbor connecting vectors, with the sums running over all neighbors of each kind, and **k** is the Bloch momenta. *H*_0_ is diagonalized analytically, from which we obtain the energy bands ϵ_*CB*/*VB*_(**k**), and the eigenstates |*u*_*CB*/*VB*_(**k**)⟩, of the valence (VB) and conduction bands (CB). From |*u*_*CB*/*VB*_(**k**)⟩ the transition dipole (**d**_*nm*_^**k**^ = *i*⟨*u*_*n*_(**k**)|*∂*_**k**_|*u*_*m*_(**k**)⟩) and momentum matrix elements (**p**_*nm*_^**k**^ = ⟨*u*_*n*_(**k**)|*∂*_**k**_ *H*_0_(**k**)|*u*_*m*_(**k**)⟩) are analytically evaluated (similar to the approach employed in refs 20 and 55 with the gauge choice specified in ref 59).

The interaction of an intense femtosecond laser pulse with graphene electrons is captured within the SBE in the length gauge, Houston basis, and while applying the dipole approximation. These are given in atomic units by^3^

$$i\frac{d}{dt}\rho_{mn}^{k}=\left(\epsilon_{m}\left(k\left(t\right)\right)-\epsilon_{n}\left(k\left(t\right)\right)\right)\rho_{mn}^{k}+i\frac{1-\delta_{mn}}{T_{2}}\rho_{mn}^{k}-F\left(t\right)\cdot \underset{l}{\sum }\left[d_{ml}^{k\left(t\right)}\rho_{\ln}^{k}-d_{\ln}^{k\left(t\right)}\rho_{ml}^{k}\right]$$ 2

where ρ_*mn*_^**k**^ is the density matrix term connecting bands *n* and *m* at *k*-point **k**. **F**(*t*) and **A**(*t*) are the electric field and vector potential of the driving laser, respectively, which are related via *c* **F**(*t*) = −d**A**(*t*)/d*t*. Throughout the text we employ a generic elliptically polarized laser pulse of the form

$$A\left(t\right)=\frac{f\left(t\right)E_{0}}{\omega c\sqrt{1+\varepsilon^{2}}}\left[\cos\left(\omega t\right)\mathrm{x̂}+\varepsilon \;\sin\left(\omega t\right)ŷ\right]$$

with *E*_0_ the field amplitude, *c* the speed of light, ω the laser frequency, *ε* the laser ellipticity, and *f*(*t*) a temporal envelope of duration $T_{pulse}=8\frac{2\pi }{\omega }$ , yielding a full-width-half-max of ∼35 fs (see the Supporting Information (SI) for details). We apply the Peierls substitution in the Houston gauge, such that **k**(*t*) = **k** + **A**(*t*)/*c*. A phenomenological term accounting for decoherence can be added with a constant dephasing time *T*_2_ regardless of the inclusion of phononic interactions that will be next described.

These equations are solved numerically (see SI), where at each time step we compute the time-dependent current:

$$J\left(t\right)=\underset{m,n,k}{\sum }w_{k}p_{mn}^{k\left(t\right)}\rho_{mn}^{k}$$ 3

with *w*_**k**_ the *k*-point weight. Note that **J**(*t*) can be separated to intraband terms (*n* = *m* case) or interband (*n*≠*m*) terms.^60^ From the currents we obtain the HHG emission as the spectral power of the Fourier transform of *∂*_*t*_ **J**(*t*).

To contrast with the phonon-free equilibrium lattice case, we also describe HHG including phononic DOF by thermally populating the longitudinal optical (LO) and transverse optical (TO) phonon modes at Γ. The effects of these modes on *H*_0_ is 2-fold. First, by populating a phonon mode the lattice geometry slightly distorts (see illustration in Figure 1(a,c)), which is described by shifting one of the two atoms in the unit cell positions away from equilibrium by Δ**R**, while fixing the lattice vectors. This alters the TB nearest-neighbor vectors (**v**_1,*j*_). Second, the shifted lattice geometry effectively alters the hopping terms to different neighbors (with closer neighbors permitting larger hopping and vice versa). We model the changes in hopping coefficients with an exponential decaying function fitted to graphene parameters,^61^ $\overset{\sim }{t_{1}\;}\left(\Delta R\right)$ (which arises from exponentially decaying overlap integrals of localized orbitals, see SI). The resulting Hamiltonian with displacement reads

$$H_{0}^{\Delta R}\left(k\right)=\left[\begin{array}{ll} t_{2}\underset{j}{\sum }e^{-iv_{2,j}\cdot k} & \overset{\sim }{t_{1}\;}\left(\Delta R\right)\underset{j}{\sum }e^{-i{ṽ}_{1,j}\cdot k}\\ \overset{\sim }{t_{1}\;}\left(\Delta R\right)\underset{j}{\sum }e^{i{ṽ}_{1,j}\cdot k} & t_{2}\underset{j}{\sum }e^{-iv_{2,j}\cdot k} \end{array}\right]$$ 4

where **ṽ**_1,*j*_ are the nearest-neighbor vectors including displacements that are no longer of equal length. Note that second-order hopping amplitudes and vectors remain unchanged with Γ-only phonons since they are A-A and B–B sublattice connections that do not change their distance with Γ-phonon displacements. Since *H*_0_^Δ**R**^ is still 2 × 2, we analytically diagonalize it and obtain exact equations for the bands, states, and dipole and momentum matrix elements, which become functions of Δ**R**. The same SBE formalism as in the equilibrium case is applied, where the value Δ**R** is sampled from the phononic distribution and fixed during the simulation (i.e., *∂*_*t*_Δ**R** = 0). We refer to this as the static phonon approximation,^19^ which should be reasonable on ultrafast timescales. In other words, electron–phonon interactions are included only unidirectionally, with phonons acting on electrons, but not the other way around. The temperature determines the width of the distribution function, calculated per phonon polarization from the variance of the position operator in a corresponding harmonic oscillator $\sigma^{2}\left(T\right)=\frac{\hbar }{M_{c}\omega_{ph}}\coth\left(\frac{\hbar \omega_{ph}}{2k_{B}T}\right)$ , with *M*_*c*_ the mass of a carbon atom and ω_*ph*_ the frequency of the Γ phonon. For instance, at 300 K we obtain a typical value for graphene of *STD*(Δ**R**)|_300*K*_ = 0.0417 Å, amounting to ∼1.7% of the lattice parameter (i.e., rather small displacements). Separate and independent simulations are performed for many values of Δ**R** that sample static “snapshots” of the lattice, and the current from these individual simulations is coherently summed to obtain the total current that includes phonon contributions (much like recent schemes in liquid HHG^57,58^ ). The simulation is formally converged with the number of snapshots (*N*_*snap*_, see data in SI). Note that besides the phononic interactions, the *T*_2_ term is still applicable and can be tested for additional effects in HHG that might be attributed to *e*–*e* scattering or other phononic channels beyond optical, and beyond Γ.

**Figure 1:**
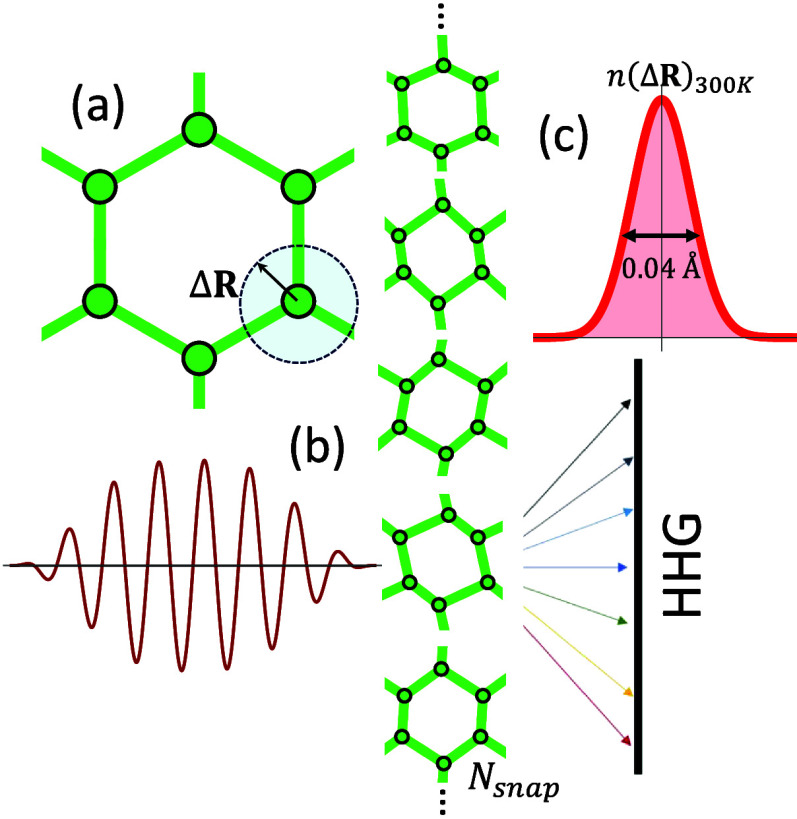
Illustration of thermal optical phonons in graphene at Γ. The graphene lattice (including phonons) is driven by an intense laser, causing HHG. (a) Equilibrium lattice (Δ**R** = 0). Possible Δ**R** displacements are indicated by the arrow and circle around one atom. (b) Series of snapshots for varying phonon-induced lattice displacements, generating static lattice configurations that are sampled with proper weights to include phononic interactions. The displacements are grossly exaggerated for illustrative purposes. (c) Exemplary phononic displacement distribution function at 300 K.

We now employ these two approaches to study HHG in graphene. Figure 2(a–f) presents HHG driven by linearly polarized pulses at typical experimental conditions (λ = 2600 nm, *I*_0_ = 1.15 × 10^12^ W/cm^2^). We observe, consistently with many prior works, that for Δ**R** = 0 (denoted as the equilibrium case), a wide HHG plateau is obtained up to ∼8 eV. This result is apparent even when including phenomenological dephasing at a reasonable timescale of *T*_2_ = 20 fs^40^ (see Figure 2(a)), which only cleans-up the harmonic spectra and symmetrizes harmonic peaks, as expected (in absence of phenomenological dephasing the spectrum is extremely noisy). On the other hand, inclusion of optical phonons (Figure 2(b)) substantially suppresses the HHG yields of nonperturbative harmonics, such that above ∼3 eV HHG is reduced by an order of magnitude or more. This effect is in agreement with multiple experiments that to date could not resolve high harmonics above ∼3.1 eV from graphene. Therefore, our theory suggests that lack of high harmonic emission from graphene might be a result of optical-phonon induced suppression (though other potential explanations could also contribute such as damage threshold, substrate effects, etc.).

**Figure 2:**
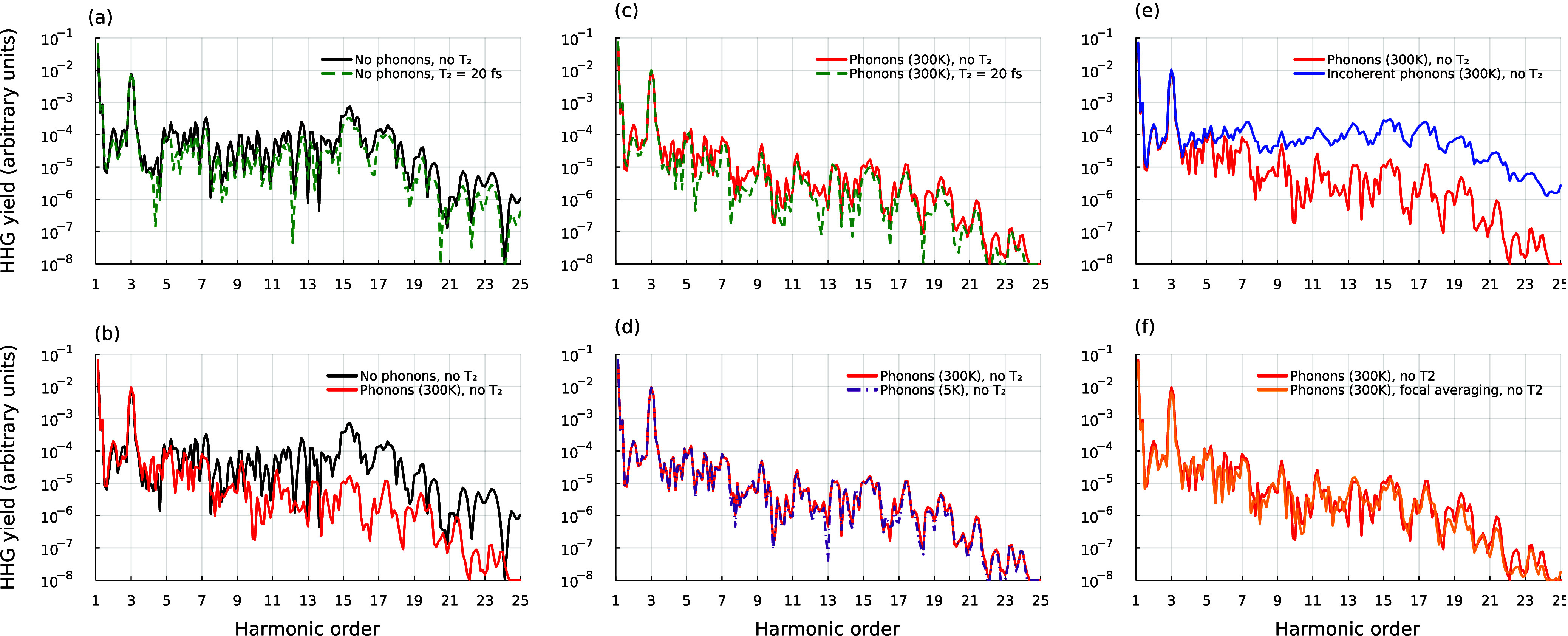
HHG spectra from graphene simulated at varying levels of theory. (a) HHG at the equilibrium case (no phononic DOF) with/without phenomenological dephasing. (b) HHG emission with phononic DOF compared to equilibrium case, showing strong suppression of the plateau and spectral cleaning. (c) HHG emission including phononic DOF and with/without phenomenological dephasing. (d) Temperature-dependence of HHG in graphene calculated including phononic DOF and without phenomenological dephasing. (e) Comparison of coherent/incoherent summations over HHG phononic snapshots. (f) HHG emission including phononic DOF with/without Gaussian focal beam averaging. Calculations performed for a peak laser power of 1.15 × 10^12^ W/cm^2^ and wavelength of 2600 nm. Data presented in logarithmic scale.

Before exploring the physical origin of this effect, we further study its temperature dependence and find that HHG is largely temperature-independent (Figure 2(d)). Physically, this occurs in graphene due to the optical phonons energy scales—at Γ phonons arise at ∼200 meV,^62^ such that at room temperature phonons are only ∼0.2*%* occupied. Thus, minor occupations dominated by zero point quantum-nuclei motion lead to the strong HHG suppression, and temperature reduction hardly changes the HHG emission. It is worth noting that under intense laser driving, it is very likely that much higher phonon occupations of optical modes occurs due to indirect heating. Generally, we notice a substantial shift in HHG yields occurring only toward ∼1500 K due to these energy scales (see SI).

We next explore the role of phenomenological dephasing introduced through the *T*_2_ term and the level of “cleanliness” of harmonic peaks. In simulations including phononic DOF a *T*_2_ term can also be added, which might account for *e*–*e* scattering channels, or other acoustic and non-Γ phononic interactions. We generally observe in simulations that HHG spectra including phononic DOF but without *T*_2_ dephasing is also substantially “cleaned”, where HHG peaks are symmetrized and noise in-between harmonics is reduced. This is somewhat analogous to HHG with addition of *T*_2_, though there still exists minor noise in-between harmonics and some asymmetric harmonic profiles observed. Figure 2(c) presents HHG spectra including both phononic DOF and a *T*_2_ term, which adds an additional minor cleaning effect, just as in the equilibrium case. In that respect, added phenomenological dephasing plays the same role even if it is combined with optical-phonon–electron interactions in the static approximation. Overall, we conclude that optical phonons in graphene have a similar impact to phenomenological dephasing in cleaning the spectrum.

Next we consider other potential origins for peak cleaning features observed in experiments. Figure 2(e) presents a comparison of coherent and incoherent summation over HHG spectra from the various snapshots employed in the phononic case (with *T*_2_ → *∞*). The correct physical procedure involves a coherent sum, as employed throughout. Nonetheless, we observe that the incoherent sum has an extremely “cleaning” effect on HHG spectra (see Figure 2(e)), while the yield suppression vanishes as expected. This motivates us to explore macroscopic beam focal averaging. HHG yields evaluated including focal averaging require the various phononic snapshot HHG spectra to be summed at two levels, both coherently within a given microscopic region, as well as incoherently from regions in the Gaussian beam that are distant from each other on length scales beyond electronic coherence,^63^ each with proper weights (see details in SI). Figure 2(f) presents this procedure, showing that both the suppression, and slightly cleaner symmetrized HHG peaks, survive (though the effect is rather small and mostly noticeable in-between harmonic peaks).

Having established prominent effects of optical phonons in HHG from graphene, we turn to analyze their physical origin. At a first step, we separate the phononic contribution to HHG emission in the inter/intraband channels. Figure 3(a) shows HHG spectra computed by including phonon DOF in the intraband emission, but fixing the interband emission to the equilibrium case, which isolates the impact of phonons on intraband harmonics. The results clearly show that interaction between phonon DOF and intraband currents does not induce a suppression. Contrarily, the same analysis performed by fixing the intraband currents and phonon-averaging over interband emission strongly suppresses HHG, but only for nonperturbative harmonics (largely above H5, see Figure 3(b)). This connects the effects to interband coherence of the electronic system. To understand why interband emission is so strongly suppressed, yet unaffected in the perturbative harmonics, we analyze HHG emission phases at a snapshot- and harmonic-resolved level. From each of the snapshots that comprise the interband phononic HHG case, we extract the harmonic emission phase, plotted as a statistical distribution in Figure 2(c–f) for select harmonics (see SI for additional data). In perturbative harmonics the phase distribution is narrow, suggesting minimal destructive interference (e.g., H5 in Figure 3(c)). In suppressed higher harmonics the phase distribution is very wide. For instance, H11 in Figure 3(d) shows very broad phase distribution that leads to massive destructive interference. In cases where specific phase values contribute dominantly, a counter peak at the opposite phase value arises, which also causes destructive interferences (e.g., H19 Figure 3(f)). The in-between harmonic region such as H16 that is symmetry-forbidden^64^ also exhibits this effect (Figure 2(e)), which allows spectral cleaning in-between orders. We coin this effect “phononic phase scrambling”, which we expect to arise generally for sufficiently occupied phonon branches. The case of graphene is unique in that here dominantly zero-point motion is sufficient to observe strong suppression, even down to few Kelvin temperatures and very small *STD*(Δ**R**). We hypothesize that this has to do with graphene’s Berry phase around the Dirac cones that carry ± π values.^65,66^ Essentially, optical phonons in graphene do not break inversion symmetry, and therefore do not open the gap or lift the nonzero Berry phase. However, even small shifts in Δ**R** break the 6-fold rotational symmetry and shifts the Dirac cones positions in *k*-space.^67,68^ We expect this causes an ambiguity of ± ϕ in HHG phases depending on if Δ**R** is positive or negative (where both branches are roughly equally populated in the harmonic phonon approximation). It remains unclear at this stage if such a mechanism is also relevant in gapped hexagonal solids, which should be a topic of future work.

**Figure 3:**
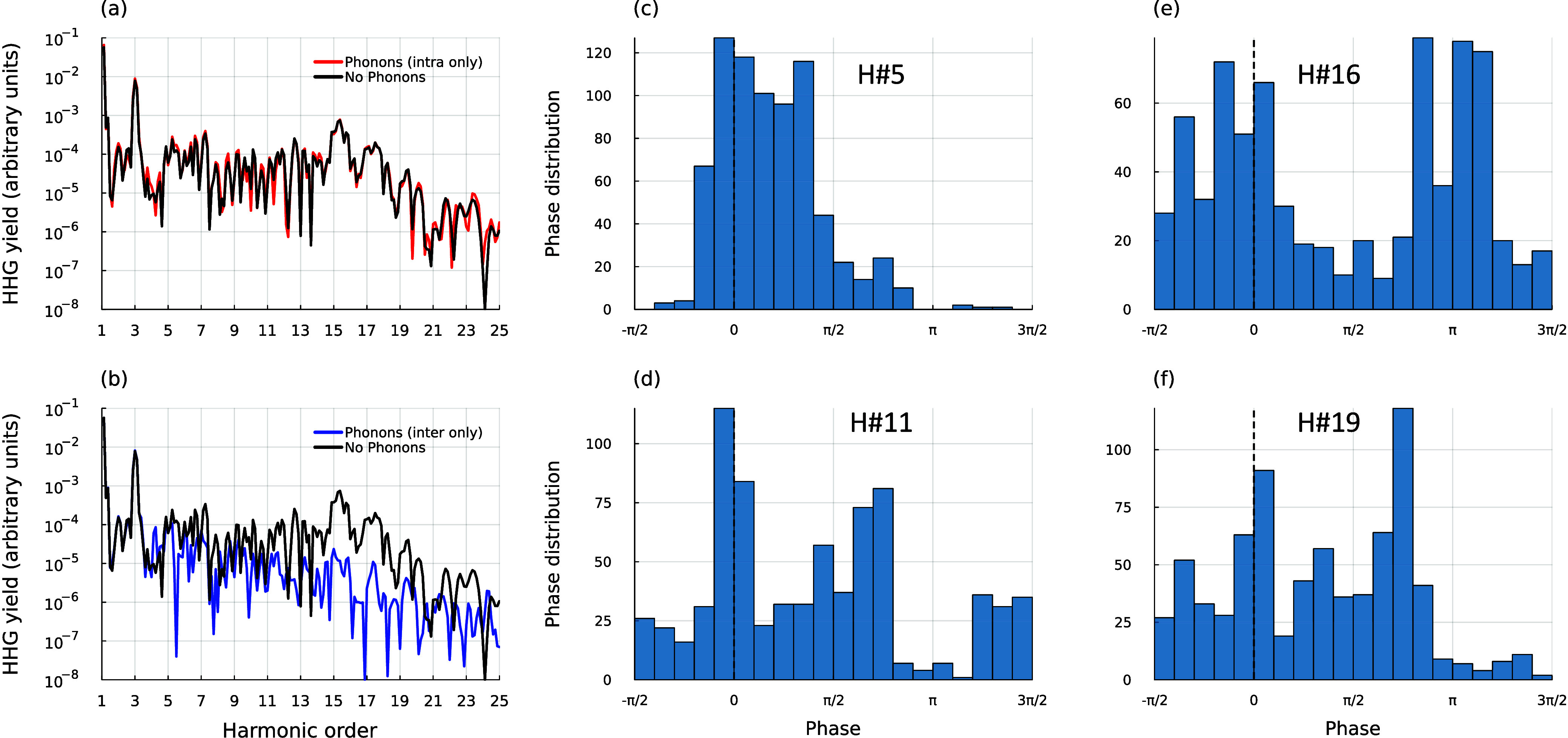
(a) HHG emission with phononic DOF included only in intraband channel, compared to the equilibrium case. (b) Same as (a) but for interband emission. (c–f) Select harmonic order phase distributions across phononic snapshots from the interband emission channel (all other harmonics are delegated to the SI). Simulations performed in similar conditions to Figure 2 without phenomenological dephasing. Zero phase in each case is offset to the equilibrium case phase (dashed black line).

We next directly explore ultrafast interband coherence dynamics. Figure 4(c–f) presents BZ-averaged values for |∑_**k**_ρ_*cv*_^(**k**)^(*t*)|, which can be explored with/without phonon DOF (phenomenological dephasing is not considered at this stage as it leads to the expected exponential decay of ρ_*cv*_(*t*) and disrupts our analysis of phonon-induced dephasing). The summation of interband coherences across the BZ is a direct part of the total interband electric current in the system (eq 3). Indeed, assuming that occupations and momentum matrix elements are fixed in time (e.g., after the laser field turns off and no phenomenological dephasing is employed), then |∑_**k**_ρ_*cv*_^(**k**)^(*t*)| fully accounts for the time-dependence in the coherence, which makes it the ideal entity to analyze for extracting coherence lifetimes. An important point in this approach that is often not addressed is that the coherence continues to evolve even in absence of a laser field due to the occupation of CB states at different *k*-points (a superposition state). Each ρ_*cv*_^**k**^ term evolves in time with the proper eigen-energy as ∼*e* ^–*i*(ϵ_*CB*_^**k**^–ϵ_*VB*_^**k**^)*t*^, which leads to a temporal dependence, as well as natural coherence decay even in absence of any dephasing channels in the simulation. However, this coherence decay in itself differs from traditional dephasing and arises from coherence leakage into higher modes as the superposition state delocalizes the wave functions in real-space. The resulting dynamics of |∑_**k**_ρ_*cv*_^(**k**)^(*t*)| is highly oscillatory, does not decay exactly to zero, and is time-linear unlike the expected exponential effect (see Figure 4(c), blue curve). As a result, we do not analyze this case, which obviously also does not cause HHG emission suppression.

**Figure 4:**
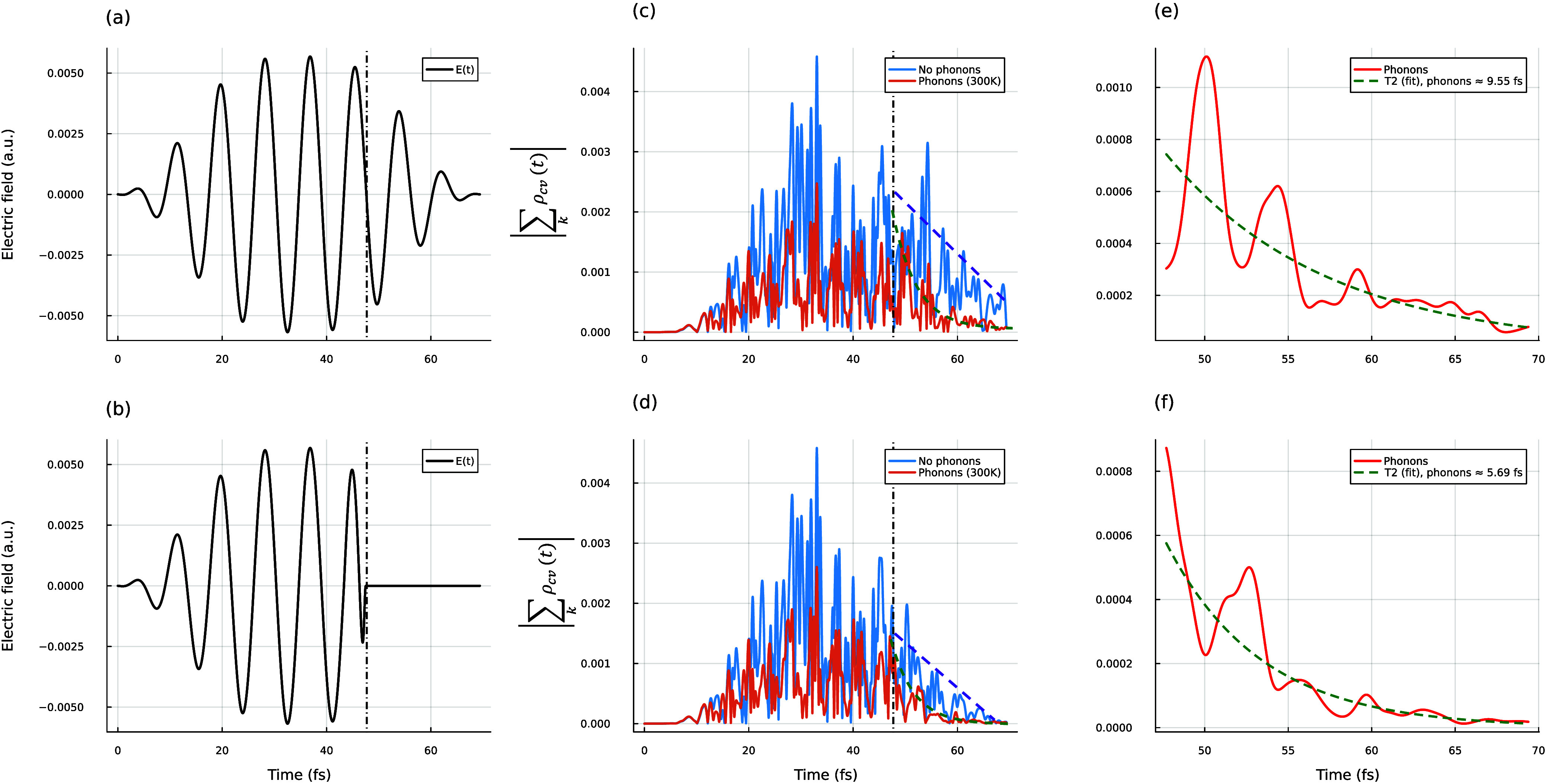
Interband coherence dynamics for BZ-averaged ρ_*CV*_(*t*). (a) Laser electric field. (b) Interband coherence dynamics with/without phonons. Vertical dashed line represents the temporal region toward the end of the pulse from which on the dynamics are fitted to an exponentially decaying function (dashed green). Linear dashed line in purple marks the nonexponential coherence leakage in the equilibrium case. (c) Same as (b) but in the temporal fitting region with the optimally fitted exponential function. (d-e) Same as (a-c) but for a case where the laser electric field is artificially halted in simulations in order to obtain a cleaner extraction of dephasing. Simulations do not include phenomenological dephasing and performed in similar conditions to Figure 3(a–f).

In Figure 4(a–c) we analyze the BZ-averaged ρ_*cv*_(*t*) dynamics including phonons in typical laser-driven conditions. The decay toward the end of the laser is fitted to an exponentially decaying function, yielding an effective dephasing time of *T*_2_^(*e*–*ph*)^ ≈ 9.55 fs. We note that this dephasing time extraction is still affected by the laser since the pulse is still “on”, driving weak transitions even in the decaying part of the envelope (which ultimately increase coherences). In order to isolate the intrinsic dephasing induced by phononic DOF without effects from the laser, we perform a set of artificial simulations where the laser is abruptly and continuously halted during simulations (see Figure 4(d)). This is achieved by adding a temporal envelope *g*(*t*) to the laser (see details in SI). Since the laser field is “off” after this moment in time, the results more cleanly represent coherence decay dynamics. Fitting ρ_*cv*_(*t*) after the pulse is turned off yields a value *T*_2_^(*e*–*ph*)^ ≈ 5.69 fs (Figure 4(e,f)). The timescales obtained here for *e*-phonon-induced dephasing under strong lasers are extremely fast, and much more rapid than those expected from *e*–*e* scattering, which would indicate they are the dominant channel of decoherence in strong-field physics. Notably, the extracted *T*_2_ is stable with increasing laser power in the strong-field regime, but increases substantially for weaker driving powers (see SI).

The directly extracted value of 5.69 fs is validated by a second indirect approach—we perform HHG simulations in the equilibrium case with phenomenological dephasing at this timescale, *T*_2_ = 5.69 fs. This indeed leads to similar magnitude suppression in HHG yields (see SI). We note that the spectrum is not exactly reconstructed, nor the coherence dynamics, indicating that phenomenological dephasing does not have the exact same effect as actual inclusion of phononic DOF. From the physical standpoint of the phase scrambling mechanism, this is clear, since phenomenological dephasing suppresses ρ_*cv*_ over time at every *k*-point. On the other hand, the suppression due to phase scrambling is localized to regions in the BZ that are modified by optical phonons (e.g., the Dirac cone), and occurs instantaneously due to destructive interferences.

Lastly, we study HHG ellipticity dependence, which in graphene is known to maximize in *ε*≠0 values^51^ (though this does not appear in all light-matter conditions^69^). Note that the mechanism for nontrivial ellipticity dependence differs from that in gapped systems like MgO which has been shown to connect with a real-space perspective.^70^ In graphene it relies on a delicate balance of the laser excitation and inter/intraband contributions.^51^ When ellipticity dependence is studied theoretically, it is often quite noisy with many side-peaks arising (see e.g. results in refs. 29,71–73 ). Figure 5 compares HHG yields from the equilibrium case to the phononic case, showing that our simulations also find HHG yields maximize at *ε*≠0. The phonon DOF “clean” the plots and reduce some of the minor side-peaks, better agreeing with experiments (e.g., for H5 here, which is in the same energy scale as H9 in ref 51.). The mechanism behind this effect is similar to the one discussed above, just that here the ellipticity tunes the HHG yield suppression. Interestingly, phononic scattering can cause minor shifts in the maximizing ellipticity value (e.g., H5 in Figure 5 shifts by Δ*ε* ∼ 0.2), which could be employed for phonon occupation spectroscopy. Qualitatively similar results are shown in the SI for another crystal orientation.

**Figure 5:**
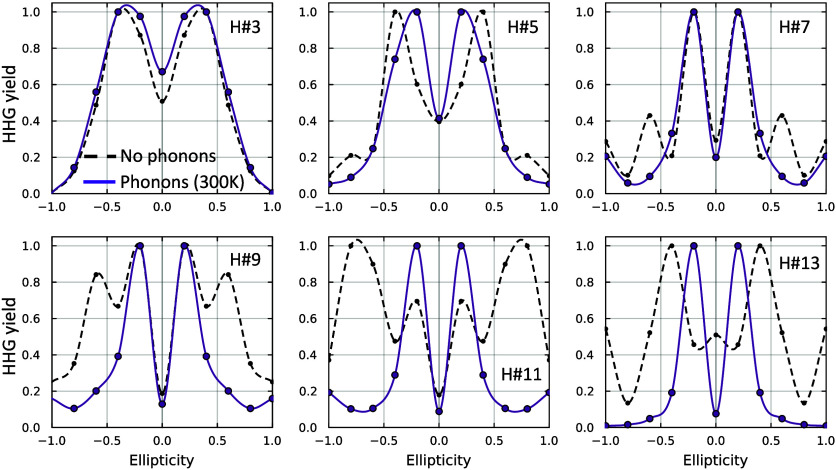
Ellipticity-dependence of normalized HHG yields for select harmonic orders with/without phononic interactions. Simulations performed without phenomenological dephasing. Note that the phononic case has suppressed HHG yield for all values of driving ellipticity, but that is not apparent due to the normalization. The elliptical major axis is chosen as the *x*-axis.

To summarize, we theoretically and numerically studied HHG from graphene, including optical-phonon–electron scattering under a static lattice approximation. We uncovered that phononic interactions can cause drastic HHG yield suppression, in agreement with previously unexplained experimental results. We studied the physical mechanism behind this suppression, showing it: (i) Does not occur in perturbative harmonics; (ii) Arises from interband current coupling to optical phonons; (iii) Occurs as a result of a “phononic phase scrambling” effect, where phonons induce wide phase variations in HHG that cause destructive interference; (iv) Is largely temperature-independent in graphene, but can be temperature-dependent in other systems depending on the phonon bands. We also showed that electron–phonon interactions directly couple to the interband coherence and effectively suppress coherence on timescales of *T*_2_ ∼ 5.7 fs, meaning the ultrafast electron–phonon interactions dominate dephasing channels in strong-field physics in solids. Lastly, we explored elliptical HHG and showed that phononic interactions smoothen harmonic ellipticity dependence, and can also shift HHG maximizing ellipticities. Thus, ellipticity-dependent HHG should be employable in HHG spectroscopies of phonon occupations.

Our results shed light on several open problems in the fields of HHG and strong-field physics. Insights obtained here can be used across material systems to both improve numerical modeling of HHG, as well as develop novel spectroscopies of electron–phonon interactions and phonon dynamics. Beyond HHG, our results should also be broadly applicable to other highly nonlinear phenomena that might couple to phonons, such as photocurrent generation,^40,74–77^ and Floquet phenomena and the issue of observing Floquet topological gaps in graphene.^78–82^

## Supplementary Material



## Notes

Additional technical details on simulations and codes, convergence data, and additional results with various laser and matter parameters (PDF)
