# Supplementary material for: Role of Ultrafast Electron-Thermal-Phonon Interactions in High Harmonic Generation and Dephasing from Graphene
Source: J Phys Chem Lett. 2026 Jul 4;17(28):7936–44. doi: 10.1021/acs.jpclett.6c01354 (PMC13383839; doi:10.1021/acs.jpclett.6c01354)
Supplement: Supplementary file 1 [file jz6c01354_si_001.pdf]

Supplementary Information: Role of ultrafast electron-thermal-phonon interactions in high harmonic generation and dephasing from graphene

Adam Herling
Technion- Israel Institute of Technology, Schulich Faculty of Chemistry, Haifa, 32000036, Israel and Technion- Israel Institute of Technology, Faculty of Physics, Haifa, 32000036, Israel

Ofer Neufeld*
Technion- Israel Institute of Technology, Schulich Faculty of Chemistry, Haifa, 32000036, Israel

This supplementary information file contains additional technical details about simulations employed in the main text, as well as additional complementary results that support our conclusions and analysis.

I. ADDITIONAL DETAILS OF SBE SIMULATIONS

SBE simulations were performed by sampling the Brillouin zone using a converged k-grid with 480×480 points spanned along the directions of the reciprocal lattice vectors. We used a 4th-order Runge-Kutta scheme to solve the time evolution of the density matrix with a converged time step of 0.2 a.u. Simulations employed the following 'super-sine' form for the laser envelope function[1]:

$$f(t) = \sin\left(\frac{\pi t}{T_{pulse}}\right)^{\pi|t/T_{pulse}-0.5|/\sigma} \tag{1}$$

with $\sigma = 0.75$ and T_{pulse} being the total pulse duration. In the decay analysis of $\rho_{cv}(t)$ in Fig. 4 in the main text, the additional smooth envelope function started at $t_{cut} = 5\frac{2\pi}{\omega}$, and had a decay time of $\tau = \frac{1}{2}\frac{2\pi}{\omega}$, with the following functional form:

$$\begin{cases} g(t) = 1 & t \leq t_{cut} \\ g(t) = \exp\left(1 - \frac{1}{1-(\frac{t-t_{cut}}{\tau})^2}\right) & t_{cut} < t < t_{cut}+\tau \\ g(t) = 0 & t_{cut}+\tau \leq t \end{cases} \tag{2}$$

where both τ and t_{cut} are synced to moments in time where the electric field vanishes.
We employed a lattice parameter for graphene at the experimental values of $a = 2.46$ Å, with hopping parameters, $t_1 = 2.72$ eV, $t_2 = 0.3$ eV. In all simulations we softened the graphene singularity of the transition dipole matrix elements at the Dirac cone to a level of $\frac{\eta}{t_1} = 10^{-3}$, where η addressed the divergence.

For simulations including phononic DOF, the hopping amplitudes were varied as: $\tilde{t_1}(\mathbf{R}_i) = t_1 \exp\left(-3\left(\frac{|\mathbf{R}_i|}{R_0} - 1\right)\right)$ where $\mathbf{R}_i$ are the displaced lattice nearest neighbor distances and R_0 is the equilibrium nearest neighbor bond length of 1.42 Å. The sampling of thermal perturbations in the phononic simulations was performed by drawing a Gaussian random variable over both Cartesian displacement components, accounting for the two Γ-point optical phonon polarizations (LO and TO). Each component was sampled independently with a standard deviation determined from the thermal variance of the displacement. The relative displacement of the atoms $\mathbf{\Delta} = \mathbf{u}_B - \mathbf{u}_A$ is calculated per phonon polarization from its canonical coordinate Q, which appears in the harmonic phonon Hamiltonian

$$H_{ph} = \frac{1}{2}\left(P^2 + \omega_{ph}^2 Q^2\right). \tag{3}$$

The canonical coordinate Q is the normal coordinate associated with the optical phonon mode obtained by diagonalizing the dynamical matrix. For the Γ-point optical phonon in graphene, the corresponding atomic displacement vectors are

$$\mathbf{u}_A = \frac{1}{\sqrt{2M_C}}\mathbf{Q}, \qquad \mathbf{u}_B = -\frac{1}{\sqrt{2M_C}}\mathbf{Q}, \tag{4}$$

* ofern@technion.ac.il

where M_C is the mass of a carbon atom. The relative atomic displacement is therefore

$$\mathbf{\Delta} = -\sqrt{\frac{2}{M_C}}\,\mathbf{Q},$$

per polarization axis. The thermal variance of the harmonic oscillator coordinate under Bose-Einstein statistics is

$$\langle Q_i^2 \rangle = \frac{\hbar}{2\omega_i} \coth\left(\frac{\hbar\omega_i}{2k_B T}\right),$$

which yields

$$\langle \Delta_i^2 \rangle = \frac{2}{M_C}\langle Q_i^2 \rangle = \frac{\hbar}{M_C \omega_{ph}} \coth\left(\frac{\hbar\omega_{ph}}{2k_B T}\right). \tag{5}$$

Using this relation between atomic displacement and temperature, and the above described random sampling procedure of the distribution functions, individual 'snapshots' of the thermally occupied lattice were drawn and the HHG spectra were simulated from each snapshot individually. The coherently summed emission from all snapshots provides HHG simulations including interactions with these phononic DOF.

The Gaussian focal beam intensity averaging procedure presented in Fig. 2(f) in the main text employed the following procedure. First, we calculated HHG spectra including full phononic snapshot coherent averaging for different laser peak powers, sampling the Gaussian beam power distribution that is described by $I(r) = I_0 \exp\big(-2(r/w)^2\big)$, with I_0 the beam peak power, w the beam waist, and r a radial coordinate away from the center. We performed 7 simulations at powers ranging from $0.85I_0$ up to I_0 with r_i sampled equidistantly. Each of the obtained HHG spectra was then incoherently summed with proper weights based on the annulus area between the corresponding radii and the next larger equidistant radius.

II. ADDITIONAL RESULTS

Here we add additional complementary results to those presented in the main text. First, Fig. S1 presents convergence data of HHG spectra in typical conditions in the phononic case vs the number of snapshots employed for sampling the optical phonon distribution (i.e. N_{snap}). Most HHG spectra were seen to converge after relatively few snapshots (order $N_{snap} \sim 300$). However, in some laser conditions slightly more snapshots were needed. Full convergence is typically obtained at $N_{snap} = 650$ regardless of the laser regime (see Fig. S1). All data in the main text employed a stricter criterion of $N_{snap} = 750$ for phononic cases.

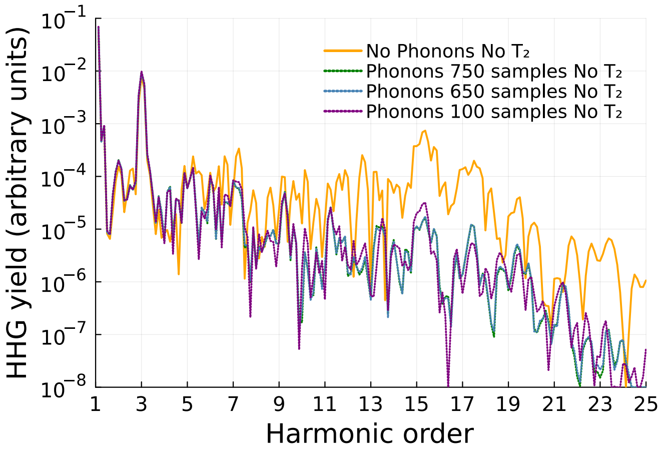

FIG. S1: Convergence of HHG emission in the phononic case with respect to number of phononic configuration sampling, N_{snap}. Simulations performed in similar conditions to Fig. 2(a-f) in main text at 300K.

Figure S2 complements the results in Fig. 2(d) in the main text, showing HHG emission in the phononic case with a very high temperature of 1500K and 2500K. Under such conditions, substantial occupation of the optical phonons is obtained, which starts impacting the HHG emission characteristics. Therefore, in the case of graphene, we only expect temperature-dependent HHG effects to start appearing in very high temperatures (where very low temperatures are

dominated by zero point motion, as discussed in the main text). However, we also note that in practice the laser pulses are likely to substantially occupy optical phonon modes during the electron-driven dynamics due to direct pumping as well as indirect energy transfer, which might lead to conditions in experiments being closer to the high-temperature conditions simulated here. Moreover, at such high temperatures, other phonon modes beyond Γ and acoustic should also contribute to the dynamics.

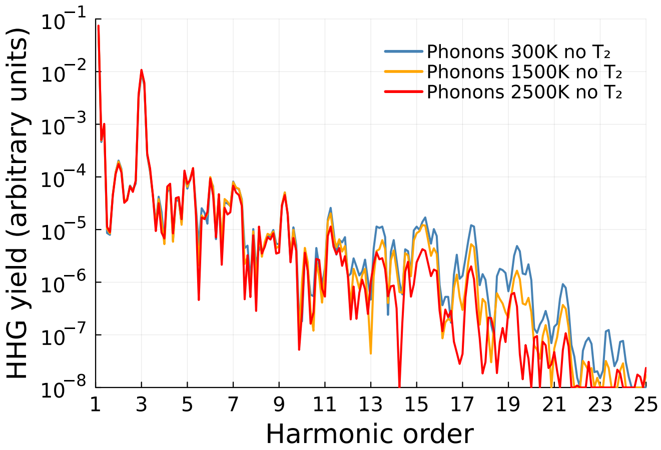

FIG. S2: HHG temperature dependence from graphene in high temperature conditions. Simulations performed in similar conditions to Fig. 2(d) in main text.

Figure S3 complements the results in Fig. 4(d,f) in the main text, extracting the effective dephasing T_2 for different laser intensities. The resulting dependence is plotted in Fig. S4, from which it can be seen that the extracted dephasing time is relatively stable in the strong field regime. However, it substantially increases at lower driving power where the coherence no longer exponentially decays (also making the fitting procedure itself less accurate).

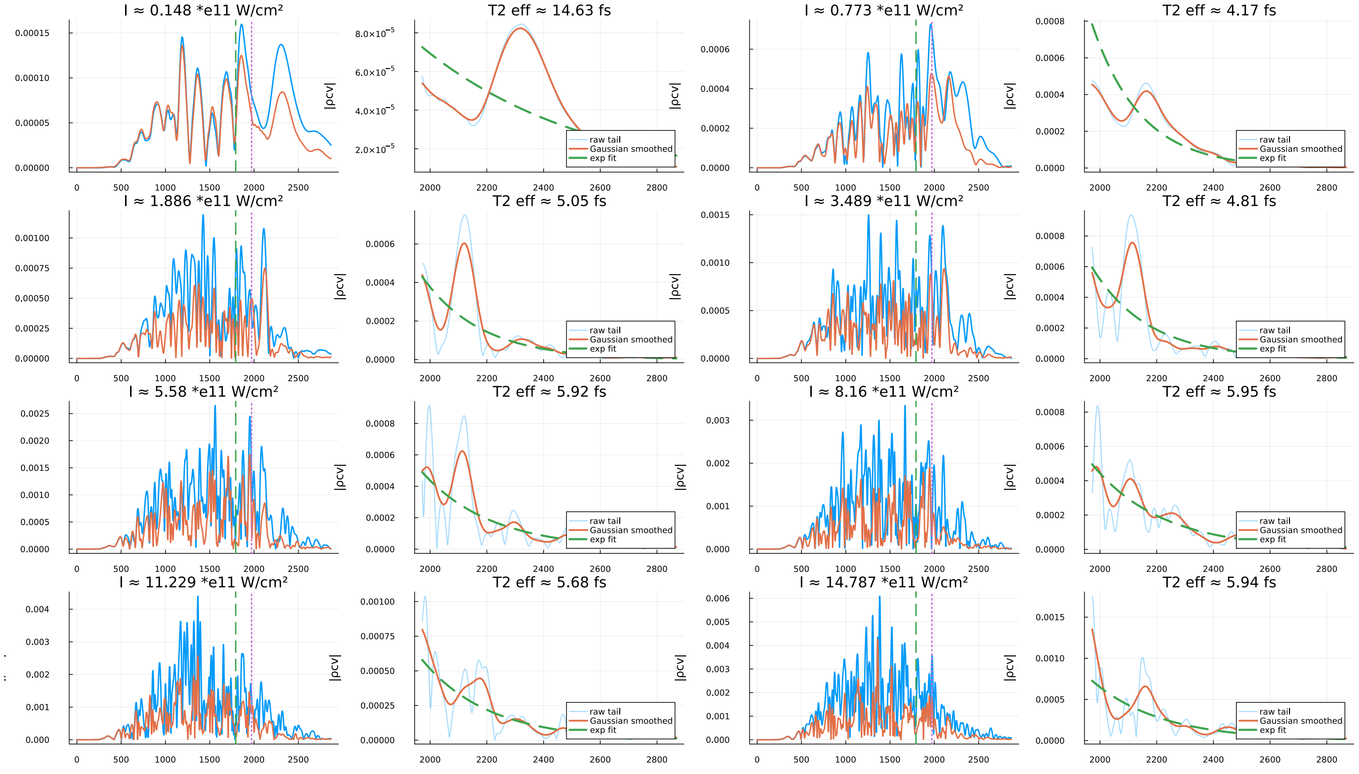

FIG. S3: T_2 extractions for different laser intensities. Method and other simulation parameters identical to Fig. 4(d,f) in main text.

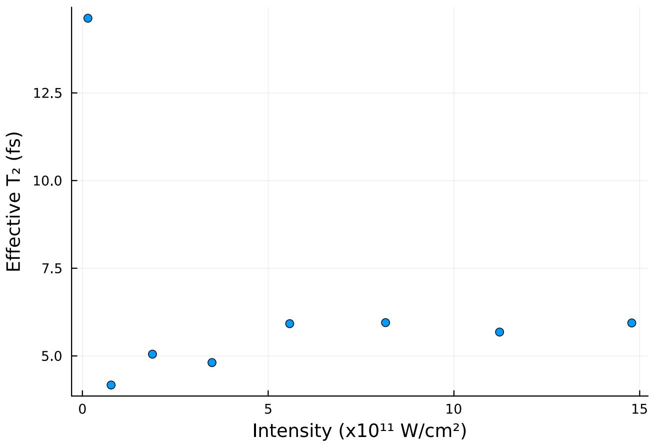

FIG. S4: Extracted effective T_2 as a function of laser intensity from data in Fig. S3

Next, we present in Fig. S5 all harmonic phase distributions across snapshots for the interband HHG channel (with intraband emission fixed at the equilibrium case). This complements Fig. 3(c-d) in the main text that only presented select harmonic orders. The phase distributions are very wide for all harmonics above 5th order, promoting destructive interferences, as discussed in the main text. The contribution of each snapshot to a bin representing a phase range $(\theta-\varepsilon, \theta+\varepsilon)$ in the histogram for a harmonic frequency H is given by $|\tilde{J}_{snap}(H)|$ for every snapshot satisfying $\arg(\tilde{J}_{snap}(H)) \in (\theta-\varepsilon, \theta+\varepsilon)$ where $\tilde{J}_{snap}(\omega)$ is the Fourier transform of the total current of the given snapshot. For the sake of numerical consistency, $\tilde{J}_{snap}(H)$ is taken to be the average of $\tilde{J}_{snap}(\omega)$ over the three frequencies closest to H.

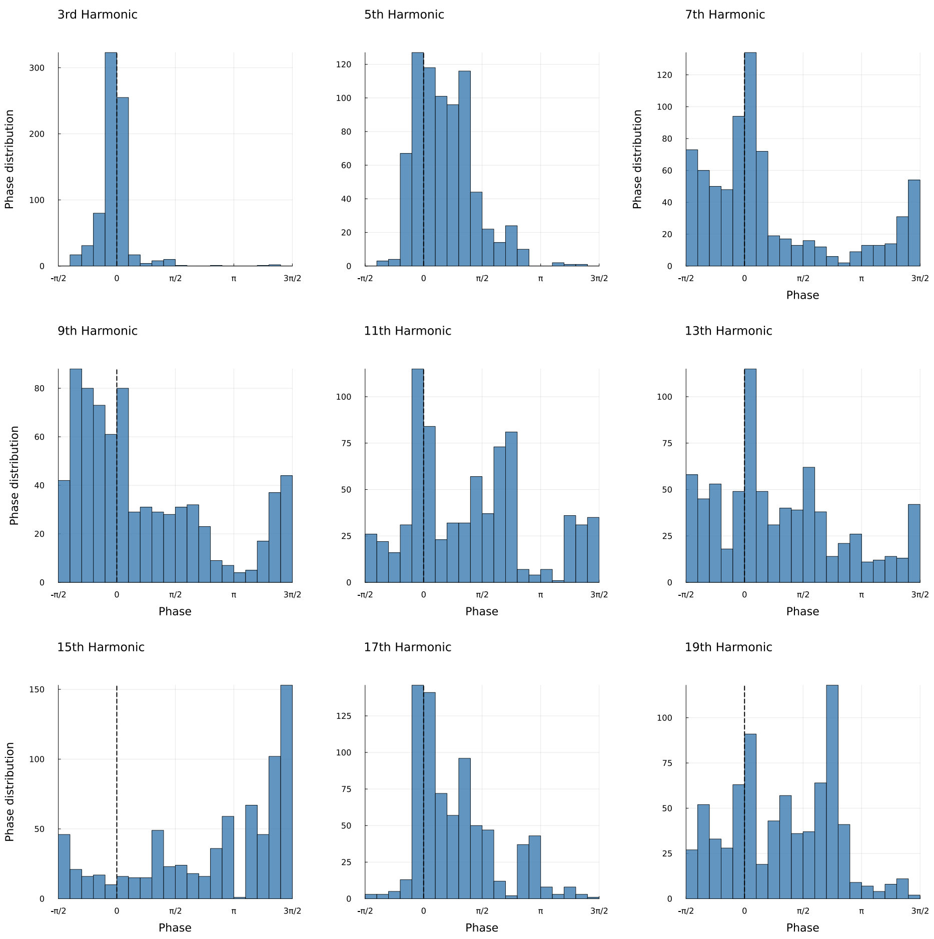

FIG. S5: Phononic phase scrambling effect in interband HHG. Histograms of harmonic phases across snapshots are presented for all odd harmonic orders, complementing select harmonic orders presented in the main text in Fig. 3(c-d) (identical simulation methodology and conditions are employed here).

Figure S6 presents HHG yields in similar conditions to Fig. 1(a,b) in the main text (equilibrium case), but with even shorter phenomenological dephasing times of $T_2 = 5.69$ fs for the equilibrium case (which is precisely the timescale obtained independently from fitting the interband coherence in the phononic case in Fig. 4(f) in the main text). This yields a very substantial suppression of HHG yields that mimics the results of the simulation that includes phononic DOF. This result therefore constitutes a second independent (and indirect) approach that validates this dephasing timescale in graphene induced by ultrafast electron-optical-phonon interactions. Notably, the spectrum is not exactly reconstructed, meaning such phenomenological terms only roughly mimic yield suppression (see discussion in the main text).

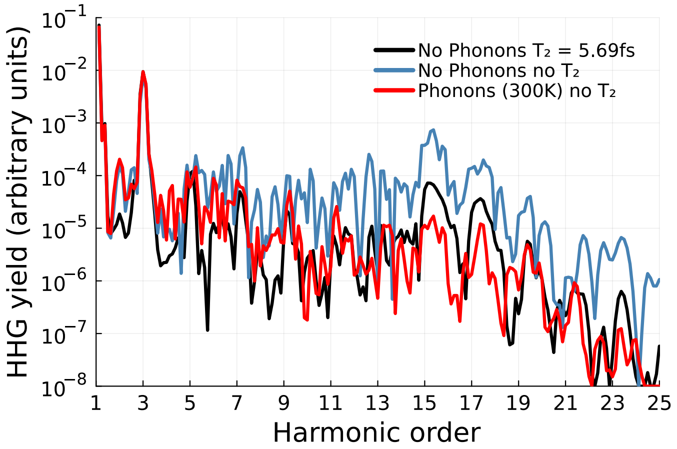

FIG. S6: HHG yields comparing the phononic case with the equilibrium case with phenomenological dephasing set to $T_2 = 5.69$ fs, which yields similar magnitudes of HHG suppression. Simulations performed in similar conditions to Fig. 1(a,b) in the main text.

Lastly, Fig. S7 presents similar ellipticity data to that shown in Fig. 5 in the main text and in the same conditions, but with the driving laser elliptical major axis along the y-axis. Qualitatively similar behavior is observed.

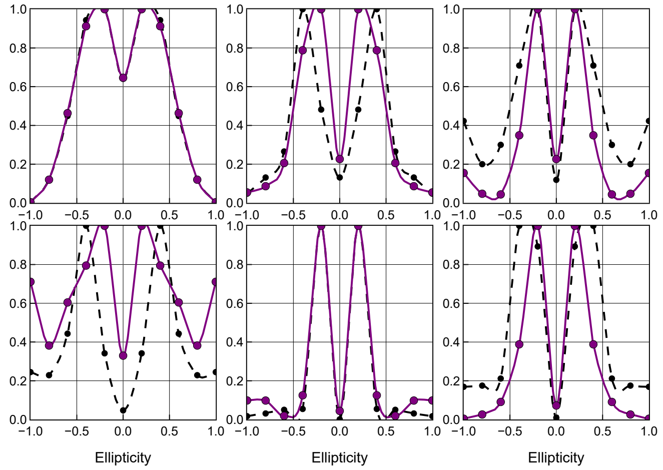

FIG. S7: Ellipticity-dependence of normalized HHG yields for select harmonic orders with/without phononic interactions in similar conditions to Fig. 5 in the main text, but with the major elliptical axis along the y-axis.

[1] O. Neufeld and O. Cohen, Background-free measurement of ring currents by symmetry-breaking high-harmonic spectroscopy, Phys. Rev. Lett. **123**, 103202 (2019).
